# Supplementary material for: Small design modifications can improve the primary stability of a fully coated tapered wedge hip stem
Source: PLoS One. 2024 Apr 17;19(4):e0300956. doi: 10.1371/journal.pone.0300956 (PMC11023602; doi:10.1371/journal.pone.0300956)
Supplement: Attachment — Submitted filename: Comments.pdf [file pone.0300956.s003.pdf]

Dear authors,

Thank you very much for your responses and for taking the time to edit the paper as well as discuss the comments in detail.

I don't have anything else to add to most of the points. Please find below further comments that require further clarification and discussion.

Response 7:

Thank you for your response. It is way more transparent now. Nevertheless, I think the following statement needs to be still discussed in more detail, including potential limitations:

"A distance of 70 mm between embedding and templated stem tip position was chosen to achieve a comparable bending situation independent from implant length [42]."

In the case of larger femurs, one should observe a larger stem tip to the distal femur, increasing the bending distance. This might have consequences regarding the stiffness of the whole system, which won't be visible in this study conducted with a standardized bending distance. Therefore, I think these consequences and their impact on the results of this study need to be discussed in greater depth.

Response 9:

Thank you for your response. Although the average maximum peak force might be relevant to forces transferred to the femur (or deformation energy of the femur), it is also influenced by other factors, such as the impaction speed or the dynamic motion of the stem. For example, It is possible to see high initial peak values in both cases where the stem undergoes rather a large dynamic movement and does not deform the femur or stay in its position (final strokes) and transfer all the loading to the femur contribution more significantly to initial stability or the fracture risk. From this perspective, the choice of the averaged maximum peak force should be discussed in greater detail. For example, some other similar studies are using integrated force values for similar types of evaluations:

https://doi.org/10.1016/j.clinbiomech.2020.105006
https://doi.org/10.1016/j.jmbbm.2019.103535

It would add value to the study if a metric/parameter/criteria could be used that is directly related to the energy absorbed by the femoral deformation.

Response 10:

Thanks for your response and extending the discussion. However, if the segmentation plays an important role, then the HU threshold used during the segmentation needs justification.
Do you have a reference for the used values (not visible in the current version)? If not, I recommend including sensitivity analysis to quantify any possible influence on the main conclusions (i.e., given the low taper angle, small deviations in geometry (+- 0.4 mm) should yield large differences in the force-displacement response of the stem during the insertion).

Response 13:

Thank you for your response. Regarding the "including the signal as a figure since we feel that it does not add any further information to images shown elsewhere [38, 39]." When we look at the references, one of them reports ex-vivo results for ceramic head insertion, and the other one is a

parametric study analyzing possible sensitivities of the hammering and stem insertion process based on an analytical approach. So, none of these studies cover the hammering force processing of an ex-vivo stem insertion. Therefore, I believe showing one example of the raw data and how the forces were analyzed would help the reader to understand and make scientific judgments based on your results.

Additional comment:

In figure 4, it can be seen a cerclage, probably for intraoperative fracture mitigation, was applied. This was not mentioned in the manuscript. A cerclage prevents fracture because it provides circumferential support, which also influences the circumferential stiffness. These changes might influence the insertion force measurements and need to be discussed.
