# Supplementary material for: Small design modifications can improve the primary stability of a fully coated tapered wedge hip stem
Source: PLoS One. 2024 Apr 17;19(4):e0300956. doi: 10.1371/journal.pone.0300956 (PMC11023602; doi:10.1371/journal.pone.0300956)
Supplement: Attachment — Submitted filename: Response to Reviewers_no2.docx [file pone.0300956.s004.docx]

# Responses to the Reviewer Comments #2

We would like to thank the editor and the reviewers for the efforts they have invested in the second round of the review and the helpful comments to improve the manuscript. We hope that our replies help to clarify any open questions.

# Reviewer #1:

## General Comment – Part A

Dear authors,
Thank you very much for your responses and for taking the time to edit the paper as well as discuss the comments in detail. I don’t have anything else to add to most of the points. Please find below further comments that require further clarification and discussion.

## Response A

Thank you for the follow up questions. We again split the comments up and addressed each of them separately. New text is highlighted in red while black text indicates parts of the original submission.

Response 7:

Thank you for your response. It is way more transparent now. Nevertheless, I think the following statement needs to be still discussed in more detail, including potential limitations:

“A distance of 70 mm between embedding and templated stem tip position was chosen to achieve a
comparable bending situation independent from implant length [42].”

In the case of larger femurs, one should observe a larger stem tip to the distal femur, increasing the bending distance. This might have consequences regarding the stiffness of the whole system, which won’t be visible in this study conducted with a standardized bending distance. Therefore, I think these consequences and their impact on the results of this study need to be discussed in greater depth.

## Response 7:

Thank you for your Response. We agree with you, this is always an issue with bone specimens. We have added an additional discussion to lines 327 - 331:

“A distance of 70 mm between embedding and templated stem tip position was chosen independent from implant length and size to achieve a comparable bending situation [43]. The stem length varies between the stem designs as well as between the stem sizes. As a result, the system stiffness is not identical for the different specimens, which might alter the reaction of the system to applied forces during cyclic loading.”

Response 9:

Thank you for your response. Although the average maximum peak force might be relevant to forces transferred to the femur (or deformation energy of the femur), it is also influenced by other factors, such as the impaction speed or the dynamic motion of the stem. For example, It is possible to see high initial peak values in both cases where the stem undergoes rather a large dynamic movement and does not deform the femur or stay in its position (final strokes) and transfer all the loading to the femur contribution more significantly to initial stability or the fracture risk. From this perspective, the choice of the averaged maximum peak force should be discussed in greater detail. For example, some other similar studies are using integrated force values for similar types of evaluations:

[I] https://doi.org/10.1016/j.clinbiomech.2020.105006
[II] https://doi.org/10.1016/j.jmbbm.2019.103535

It would add value to the study if a metric/parameter/criteria could be used that is directly related to the energy absorbed by the femoral deformation.

## Response 9:

Thank you for this further clarification. Impaction with a hard-on-hard contact as in orthopedics is a complicated situation. We can see your point of giving more information on the impaction process as it can vary not only between specimens but also over the implantation process of a single stem.

We will include the following figure in our manuscript to give an example for a typical signal (line 182):

“Figure 4: Typical force signal for a single mallet stroke with the maximum force marked.”

The following sentence will reference to it (lines 179-181):

“Each cavity preparation with the final broach and each stem implantation required a minimum of eight mallet strokes. The maximum peak force was calculated as the mean of the maxima of the last eight strokes applied. Figure 4 depicts a typical single mallet stroke and its maximum [38,39].“

Based on your idea, we have conducted an analysis of the integrated force values. The method was adopted from the methodology introduced by the papers you referenced as well as their sources ([III] https://doi.org/10.1371/journal.pone.0166778, [IV] https://doi.org/10.1115/1.4029505

and [V] https://doi.org/10.1177/095441191455243).

The force signal is integrated from the time point when it first exceeds 300 N. The global maximum of a single stroke is included and the integration ends when the force signal falls below 300 N. The threshold of 300 N was taken to clearly identify the signal against the noise. Afterwards the signal was divided by the timespan of the integration to find an averagely applied force per stroke. This new parameter is called impaction momentum (see I to II). We again calculate the mean for the last eight strokes.

During broaching, the impaction momentum was similar for both designs (Corail: 2198.98 N (Q1 – Q3: 1968.2 – 2585.1 N), Emphasys: 2652.22 N (Q1 – Q3: 2066.8 – 3561.4 N), p = 0.116). During implantation, however, the impaction momentum was higher for the Emphasys stems (Corail: 3265.57 N (Q1 – Q3: 2575.7 – 3603.8 N), Emphasys: 3916.86 N (Q1 – Q3: 3852.5 - 4581.6 N), p = 0.046).

Figure 1: Impaction momentum during (a) broaching and (b) implantation for both stem designs.

When now the results of the maximum peak forces during broaching are compared to the newly calculated impaction momentum (fig. 1 a) the significantly higher peak forces are only trend wise shown in the impaction momentum. This result is mirrored for the impaction momentum during implantation (fig. 1 b) were now the impaction momentum is showing a significant difference while the maximum peak force is only showing a trend towards higher values for the Emphasys stem. There is additionally a high correlation between the maximum peak forces during broaching and the impaction momentum during broaching (Corail: R² = 0.889; p = 0.005; Emphasys: R² = 0.785; p = 0.019) with significant results for the implantation, too (Emphasys: R² = 0.889; p = 0.005). Only the results during implantation for the Corail (R² = 0.236; p = 0.236) were again influenced by the two Dorr type C femurs.

With these results we conclude that calculating the impaction momentum yields more information on the stroke dynamics. In this specific study the trends of the results however, are similar: the peak forces as well as the impaction momentums tend to be higher for the Emphasys stems. We will keep in mind for further studies to include more of the stroke dynamics. Especially including seating curves will be one of the goals for upcoming studies and we thank you for the idea to include it. We hope you can accept our point of view. For further clarification in our manuscript we have added the following sentence to the discussion (lines 351 - 355):

“It is possible that other factors, such as the bone densification which was not investigated [18], might influence the impaction forces. Recording seating curves can give further information on the seating behavior which might be influenced by the component’s geometry. This might affect the forces and will be investigated in further studies. The analysis of the stroke dynamics similar to [45,46] might increase the understanding of the impaction process.“

45.	Dubory A, Rosi G, Tijou A, Lomami HA, Flouzat-Lachaniette CH, Haïat G. A cadaveric validation of a method based on impact analysis to monitor the femoral stem insertion. J Mech Behav Biomed Mater. 2020; 103:103535.

46.	Albini Lomami H, Damour C, Rosi G, Poudrel AS, Dubory A, Flouzat-Lachaniette CH, Haiat G. Ex vivo estimation of cementless femoral stem stability using an instrumented hammer. Clin Biomech. 2020; 76:105006.

Response 10:

Thanks for your response and extending the discussion. However, if the segmentation plays an important role, then the HU threshold used during the segmentation needs justification.
Do you have a reference for the used values (not visible in the current version)? If not, I recommend including sensitivity analysis to quantify any possible influence on the main conclusions (i.e., given the low taper angle, small deviations in geometry (+- 0.4 mm) should yield large differences in the force-displacement response of the stem during the insertion).

## Response 10:

Thank you for your Response. In Kluess et al. it is stated that bony material can be identified from HU values starting at 200 – 250 HU [39]. In combination with Garg et al. who identified a value of 275 HU to be the threshold [38], a value of 250 HU was chosen similar to Gargiulo et al. [40] and applied during the segmentation.

We will add the following references for the HU threshold (lines 183 -185):

“For the contact analysis the femur models were segmented from the CT-scans of the femurs with prepared cavities based on a threshold of 250 to 2000 mgHA/cm³ to include trabecular as well as cortical bone (Fig 5a) [38-40].”

38.	Garg A, Deland J, Walker PS. Design of Intramedullary Femoral Stems Using Computer Graphics. Eng Med. 1985;14(2):89–93.

39.	Kluess D, Souffrant R, Mittelmeier W, Wree A, Schmitz KP, Bader R. A convenient approach for finite-element-analyses of orthopaedic implants in bone contact: Modeling and experimental validation. Comput Methods Programs Biomed. 2009; 95(1):23–30.

40.	Gargiulo P, Pétursson T, Magnússon B, Bifulco P, Cesarelli M, Izzo GM, Magnúsdóttir G, Halldórsson G, Ludvigsdóttir GK, Tribel J, Jónsson H. Assessment of Total Hip Arthroplasty by Means of C omputed T omography 3 D Models and Fracture Risk Evaluation. Artif Organs. 2013; 37(6):567–73.

We also appreciate the second aspect of your response and would like to refer to lines 363 - 364 where we addressed the different shapes of the stems in connection to the contact area. However, the forces were more affected by the canal shape during broaching. We are sorry, but we cannot make any statements about the displacement during implantation as this was not recorded (please see our answer to Response 9). We will keep it in mind for further experiments.

“The higher contact areas achieved with the Emphasys stems can be explained by the wider proximal design of the modified stem.”

Response 13:

Thank you for your response. Regarding the “including the signal as a figure since we feel that it does not add any further information to images shown elsewhere [38, 39].” When we look at the references, one of them reports ex-vivo results for ceramic head insertion, and the other one is a parametric study analyzing possible sensitivities of the hammering and stem insertion process based on an analytical approach. So, none of these studies cover the hammering force processing of an ex-vivo stem insertion. Therefore, I believe showing one example of the raw data and how the forces were analyzed would help the reader to understand and make scientific judgments based on your results.

## Response 13:

Thank you for your response. You are right and the studies we referenced to do not show signals that were recorded in a similar set-up or with a similar scope. Therefore, we will include a figure of our signal to improve the understanding and possibility to judge for the reader. Please see our answer to your response 9.

Additional comment:

In figure 4, it can be seen a cerclage, probably for intraoperative fracture mitigation, was applied. This was not mentioned in the manuscript. A cerclage prevents fracture because it provides circumferential support, which also influences the circumferential stiffness. These changes might influence the insertion force measurements and need to be discussed.

## Response

Thank you for this comment, we are sorry, for the confusion we might have caused with this but we did not use any cerclage during the experiments. In figure 4 a rubber band can be seen which was used to attache tags identifying the femurs during the CT-scans. We moved these rubber bands distally during the experiments (see figure 3). They were not used for any stabilization of the femur and could not change the results. However, we added the following sentence to the description (lines 197- 199):

Figure 5: […] (a) a CT-scan of the prepared bone with cavity and rubber band tag to distinguish between femurs, (b) […]

We thank you for your really thorough revision of our manuscript!

# Reviewer #2

## General Comment

Small design modifications can significantly enhance the primary stability of a fully coated tapered wedge hip stem. Through meticulous adjustments in geometry or material composition, the stem can achieve optimal fixation within the bone, reducing the risk of implant loosening or migration. Enhancements may include refining surface texture to promote osseointegration, optimizing stem length or diameter for better fit, or incorporating features to distribute load more evenly. Such modifications bolster initial stability, crucial for successful long-term implant function and patient mobility. Even seemingly minor alterations can yield substantial improvements, underscoring the importance of precision in orthopedic implant design.

Good work

## Response

Thank you again for conducting the review to our manuscript and the positive feedback.

Further changes:

- using the newest EPRD Report:
  Grimberg A, Lützner J, Melsheimer O, Morlock M, Steinbrück A. EPRD Jahresbericht 2023 [Internet]. DE: EPRD Endoprothesenregister Deutschland; 2023 [cited 2024].
- Grammatical error in lines 320 – 321:
  “These results do not only apply to femurs of Dorr type A but to all Dorr categories, as in this study all Dorr types were used.”
- Figure numbering from figure 4 upwards was adapted to include the new figure 4. In old figure 7 and 8 the correlation coefficients were corrected to the ones in the text. All figures were reuploaded again with a higher quality without changes of the contents other than already mentioned.
